# Whole genome sequencing of macrolide resistant *Streptococcus pneumoniae* serotype 19A sequence type 416

**DOI:** 10.1186/s12866-020-01909-1

**Published:** 2020-07-25

**Authors:** Petra Spanelova, Vladislav Jakubu, Lucia Malisova, Martin Musilek, Jana Kozakova, Costas C. Papagiannitsis, Ibrahim Bitar, Jaroslav Hrabak, Annalisa Pantosti, Maria del Grosso, Helena Zemlickova

**Affiliations:** 1 Centre for Epidemiology and Microbiology, National Institute of Public Health, Prague, Czech Republic; 2 Department of Clinical Microbiology, Faculty of Medicine and University Hospital, Charles University, Hradec Kralove, Czech Republic; 3 Faculty of Medicine, Biomedical Center, Charles University, Plzen, Czech Republic; 4 Department of Infectious Diseases, Istituto Superiore di Sanità, Rome, Italy; 5 Department of Laboratory Medicine, Third Faculty of Medicine, Charles University and Kralovske Vinohrady University Hospital, Prague, Czech Republic

## Abstract

**Background:**

The resistance of *Streptococcus pneumoniae* to macrolides is becoming an increasingly important issue and thus it is important to understand the genetics related to adaptation of this species to the widespread use of antibiotics in Europe. The 58 isolates of *S. pneumoniae* belonging to sequence type (ST) 416 and serotype 19A and to several different phenotypes originated from Italy, Portugal and Czech Republic were thus sequenced on Illumina MiSeq. The aim of the study was to describe genetical origine of isolates, investigate their macrolide resistance and suggest reasons for spread of ST416 in the Czech Republic.

**Results:**

Investigation of genes associated with serotype determined serotype switch between 15B and 19A serotypes and core genome multilocus sequence typing (cgMLST) confirmed the origine of concerned isolates in Netherlands^15B^-37 clone. Inspected genomes proved variability of genes associated with the macrolide resistance even within closely genetically relative isolates.

**Conclusions:**

Participation of 19A/ST416 on the spread of Netherlands^15B^-37 is accompanied by serotype switch between 19A and 15B serotypes and with acquisition of genes involved in macrolide resistance to the clone that was originally macrolide susceptible. There is evident tendency to interchanging and modifications of these and surrounding genes, that could lead to accelerate spreading of this sequence type in regions with high macrolide consumption.

## Background

*Streptococcus pneumoniae* is a leading cause of community-acquired pneumonia, meningitis, acute otitis media, acute sinusitis, and bacteremia. Pneumococcal infections are mainly prevalent in infants, elderly, and immunocompromised patients. It is estimated that 0.8 million children die every year from pneumonia in which pneumococcus is considered the major pathogen [1]. The main reservoir of *S. pneumoniae* in humans is the nasopharynx. Carriage rates are highest in children of preschool age (up to 50%), in adults the carriage rates ranges between less than 5% to more than 20% depending on factors such as age, immune status and antibiotic use [2]. Although the colonization of the nasopharynx is usually asymptomatic, carriage can progress to invasive respiratory or systemic disease [3]. Pneumococci are encapsulated, the polysaccharide capsule which protects pneumococci from phagocytosis, is considered as the major virulence factor. To date, at least 97 diverse capsular types have been described [4], although only a limited number of serotypes cause the vast majority of pneumococcal infections, some of them are also associated with antibiotic resistance.

The current prevention strategies of *S. pneumoniae* infections include the use of pneumococcal conjugate vaccines (PCV) which cover a subset of serotypes selected based on their contribution to pneumococcal infections. The first conjugated vaccine PCV7 targeting seven serotypes (4, 6B, 9 V, 18C, 19F, and 23F) was implemented in 2000 in USA [4]. The use of vaccines lead to decrease of invasive pneumococcal diseases (IPD). However, due to the limited number of serotypes included in the vaccines, an increase of occurrence of non-vaccine serotypes was reported ([4]). The serotype, which was globally associated with replacement disease after PCV7 use is 19A [5–9]. Currently, PCV10 (PCV7 plus serotypes 1, 5, and 7F) and PCV13 (PCV10 plus serotypes 3, 6A, and 19A) are used. Use of PCV10 and PCV13 led to an additional reduction in IPD caused by the additional serotypes included in the vaccines, but the cross-protection of PCV10 against vaccine-related serotypes 6A and 19A seems variable in different studies [10–12].

A substantial part of serotype 19A isolates are also resistant to antibiotics. Antibiotic resistance is in general caused by changes in core genes, or by acquisition of accessory genes, it is presumable that the later vary within clones more than the previous. Resistance to penicillin is in pneumococci caused predominantly by changes in penicillin-binding proteins situated near the capsular polysaccharide locus (*cps* locus). On the other hand, resistance to macrolides is caused by acquisition of particular genes (*erm*B, *tet*M). Distribution of clonal complexes (CC) vary in different countries, CC320 (Taiwan^19F^-14 clone) is spread worldwide, and it is the most frequent 19A clone in Asia and South America, CC199 (Netherlands^15B^-37 clone) is the predominant clone in North America and in Europe. There is a number of other less populated clones, from which the most frequent are CC695 in North America, and CC230 (Denmark^14^–32 clone) or CC156 (Spain^9V^-3 clone) in Europe [13–19].

In the Czech Republic, vaccination using PCV10 or PCV13 has been included to the routine childhood immunization programme since January 2010, both vaccines sharing the market equally. The vaccine uptake is continuously declining, from 81% vaccinated children in 2011 to 68% in 2017. In the post-PCV era, 19A isolates were found in carriage and respiratory samples and occurred also in IPD cases in all age cohorts [20]. The overall prevalence of the 19A serotype increased from 2.7% in 2010 to 9.9% in 2015, with 19A being the second most frequent invasive serotype reported in the Czech Republic [21]. Sequencing identified ST416, a double-locus variant (DLV) of the Netherlands^15B^-37 clone (CC199), as the dominant emerging genotype within serotype 19A [22]. Multilocus sequence typing (MLST) analysis of penicillin and/or macrolide resistant isolates of serogroup 19 revealed CC199 represented by ST416 in 64% of the serotype 19A isolates, other minor CCs were CC230 (Denmark^14^–32 clone), and CC193 (Greece^21^–30 clone). Participation of 19A/ST416 in the serogroup 19 was 44% in 2014 and 68% in 2017 (the second were 19F/ST1464 representing 25% of serogroup 19 in 2014 and 19A/ST320 12% in 2017 respectively) Isolates belonging to ST416 were penicillin susceptible, but erythromycin, clindamycin, and tetracycline resistant [22, 23].

Contribution of ST416 on increase of 19A serotype has been described in other European countries. In Italy, proportion of 19A isolates from IPD increased from 8% in in 2001–2003 to 17% in 2006–2009, and ST416 from 6 to 10% respectively [7, 8]. Increase of ST416 was accompanied with growth (in Italy) or introduction (in Germany) of macrolide resistance within CC199, which was originally susceptible to macrolides. According to public database of sequence types (PubMLST [24]), the isolates showing ST416 are present in Great Britain, Germany, Italy, and Spain from 2000, single isolates appeared in Australia, USA, Canada and China after year 2007.

## Results

### SNP phylogeny

Mapping of Illumina reads to the Netherlands^15B^-37 representative contigs gave consensus sequences with percentages of unambiguously calling bases in the range 91–98%. The input alignment for maximum likelihood phylogeny after the recombination sequences were removed consisted of 1,631,892 sites from which were 7367 polymorphic.

**Fig. 1:**
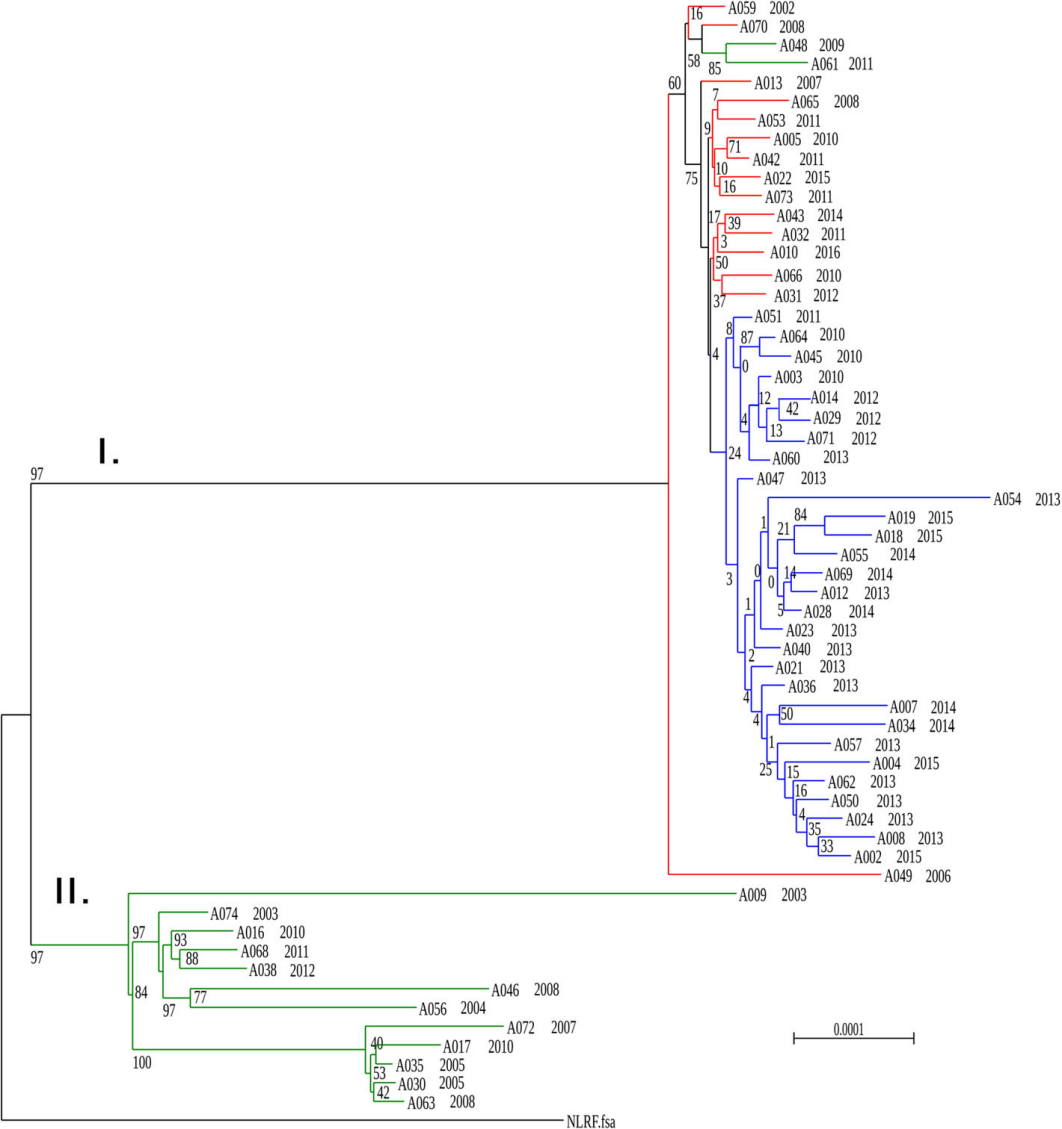
SNP phylogeny of 58 *Streptococcus pneumoniae* ST416 isolates with Netherlands^15B^-37 as reference sequence. Branches are labelled with strain numbers and year of isolation. They are coloured according to the country of origin: in red those from Italy, in green from Portugal and in blue from the Czech Republic

The dendrogram (Fig. 1) differentiated two clades, one was represented by a homogenous group of 46 isolates (Clade I), while the other included a more divergent group of 12 isolates (Clade II). There were isolates from Portugal, Italy and the Czech Republic isolated in 2002–2015 in Clade I. Four strains from Clade I were susceptible to erythromycin, clindamycin, and tetracycline, two were susceptible only to clindamycin, one only to tetracycline and the remaining isolates were resistant to erythromycin, clindamycin, and tetracycline. The topology demonstrates separations of the branches according to countries of origin. Clade II consisted of strains from Portugal isolated in 2002–2011, and it was differentiated into a few distinct sub-branches, with antibiotic profiles characteristic for each sub-branch.

### Core genome MLST

**Fig. 2:**
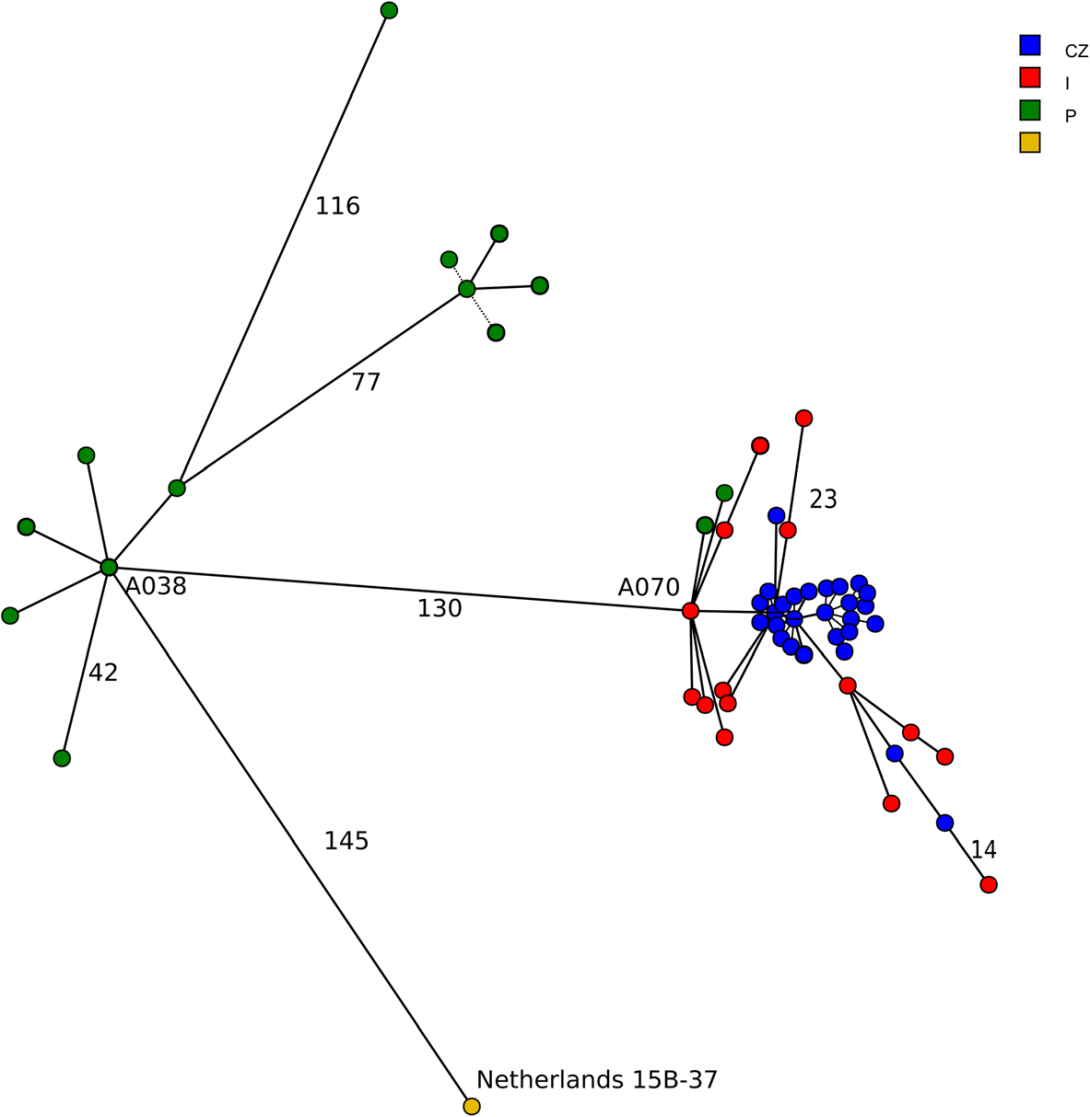
Core genome MLST of collection consisting of 58 *Streptococcus pneumoniae* ST416 isolates and Netherlands^15B^-37. Lengths of branches correspond to number of different loci. Nodes are coloured according to the country of origin: in red those from Italy, in green from Portugal and in blue from the Czech Republic

There were found 1177 groups of orthologs that were presented once in each genome. From these were 450 represented by an identical allele in each genome and 7 by different alleles in more than ¾ of genomes. cgMLST scheme was developed on basis of the rest 720 genes. Minimum-spanning tree (Fig. 2) demonstrates that Netherlads15B-37 differs from the nearest genome of Clade II in 145 loci and that differs from the nearest genome of Clade I in 130 loci. The longest distance between two genomes within a clade was 23 loci in Clade I and 116 loci in Clade II.

### Relatedness between ST416 and reference strain Netherlands15B-37

Netherlands^15B^-37 (ATCC BAA-1663) represents ST199 which is a DLV of ST416. They differ in the *aro*E allele by one single nucleotide polymorphism (SNP), and in the *xpt* allele by six SNPs. According criteria previously defined, the change in *aro*E most probably arose by a point mutation and while that in *xpt* arose by recombination [25].

De novo assemblies could not be used neither for reconstruction of *cps* locus and flanking sequences nor for transposable elements, as involved adjacent genes were rarely included in the same contig, that was probably caused by presence of repetitive sequences.

**Fig. 3:**
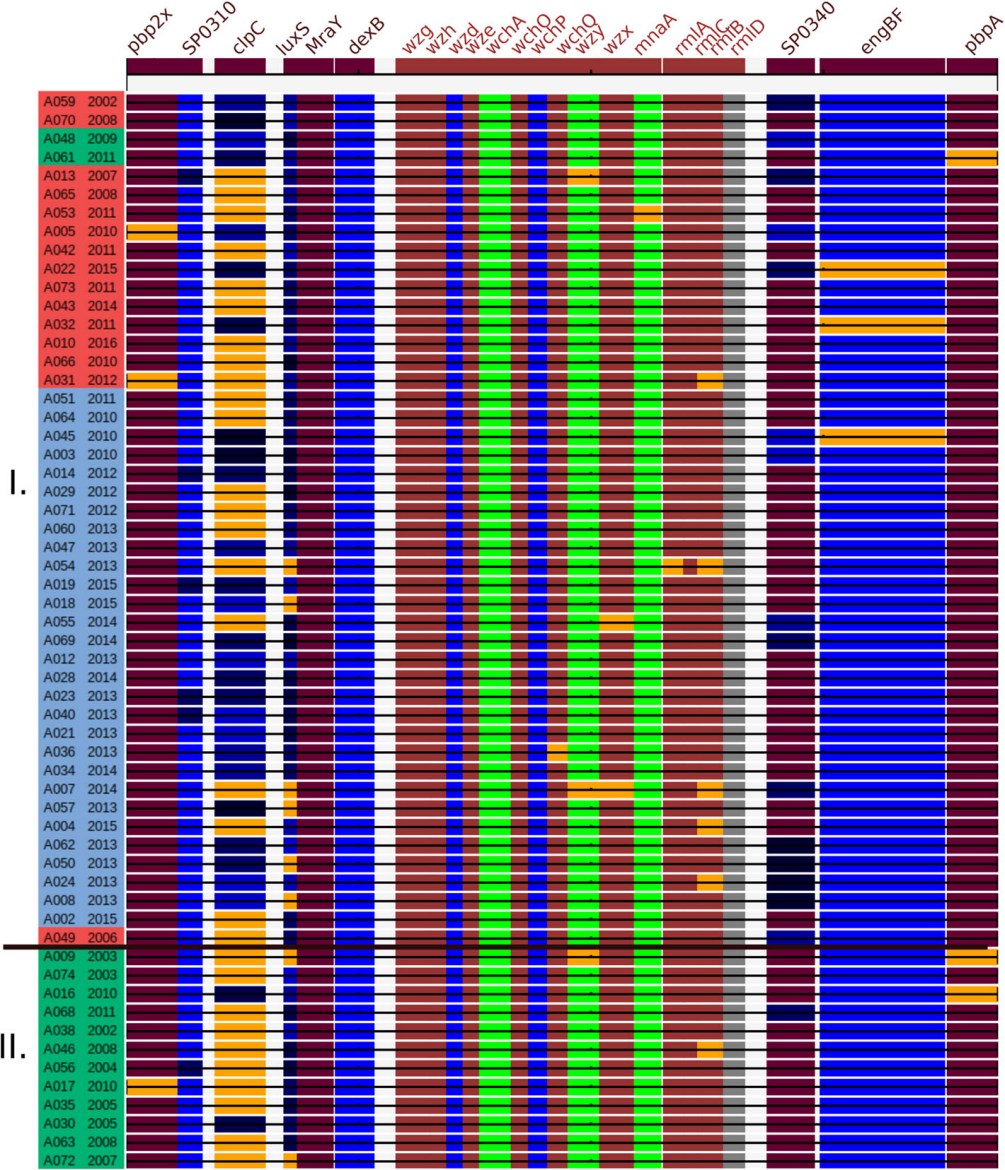
Relatedness between sequences of particular genes involved in serotype switch and the corresponding genes in references. Genome diagram compiled from assemblies with three reference sequences: region between start of *pbp*1a and end of *dex*B in Netherlands^15B^-37 (coloured in dark brown), 19A *cps* locus (coloured in light brown) and region between start of *ali*A and end of *pbp*1a (coloured in light brown). Genes with sequences identical between particular strain and corresponding gene in the genome of Netherlands^15B^-37 are coloured in dark brown, those identical to the sequences from *cps* locus in brown. (SP0310, SPAR147_310; SP0340, SPAR147_0340) Genes with sequences different to reference and to all samples in the collection are shown in orange. Genes with sequences differing to reference in one SNP same in > 1 sample are coloured in green, those differing in the same SNPs > 1 sample are represented in shades of blue, genes which were not found in particular genome are grey. Isolates are ordered and labelled likewise in Fig. 1

Detailed investigation of *cps* locus and flanking genes (Fig. 3) showed a close relationship between the 19A *cps* loci within the collection, as well as regions of high similarity to the sequences surrounding Netherlands^15B^-37 *cps* locus. Sequences of *dex*B, and MraY situated upstream the *cps* locus were identical within the collection, *dex*B was 98% identical to the Netherlands^15B^-37 representative, the other 100% identical. Sequences of remaining genes situated between *pbp*2x and the beginning of the *cps* locus had greater variability when compared to other genes from the region. The *wzg*, *wzh*, *wzd*, *wze*, *wchA*, *wchO*, *wchP,* and *wchQ* genes from the beginning of *cps* locus were identical within the collection, the remaining genes of the *cps* locus, as well as *pbp*2x, *pbp*1a, and flanking genes differed in a few isolates. Sequences of *pbp*2x, and *pbp*1a of most isolates were 100% identical with the corresponding sequences in Netherlands^15B^-37 strain, sequence of gene *eng*BF coding endo-alpha-N-acetylgalactosaminidase situated upstream *pbp*1a differed from reference in most of isolates by the same recombination positioned at the beginning of the sequence (from 1 to 1,309 bp). Sequence of oligopeptide-binding protein a*li*A were 100% identical with the corresponding sequences in Netherlands^15B^-37 strain in 42 isolates, in remaining 16 isolates were present 5 different alleles of *ali*A. High variability in particular genes indicates that these areas are changing very quickly within the clone, and those events could be associated with serotype switches.

### Genes of resistance to erythromycin, clindamycin and tetracycline

The genes coding macrolide resistance, *mef*(A)/*mel*, *mef*(E), *erm*(A) and *erm*(TR) were not found in any of the isolates belonging to ST416. Both *erm*(B) and *tet(*M) were present in genomes of 47 isolates, from these were 37 phenotypically resistant to erythromycin, tetracycline, and clindamycin, 7 were phenotypically resistant to erythromycin and tetracycline, showing inducible resistance to clindamycin. Three isolates were phenotypically susceptible to tetracycline and resistant to other two examined antibiotics. Alignments of *tet*M show deletion of a single base leading to a frameshift mutation in the beginning of gene (position 650 resp. 653) in two genomes, phenotypical susceptibility of the third is not explainable by presented methods. Sequences of *erm*(B) and *tet*(M) were not identical within the collection, there were four different alleles of *tet*(M), and three different alleles of *erm*(B).

**Fig. 4:**
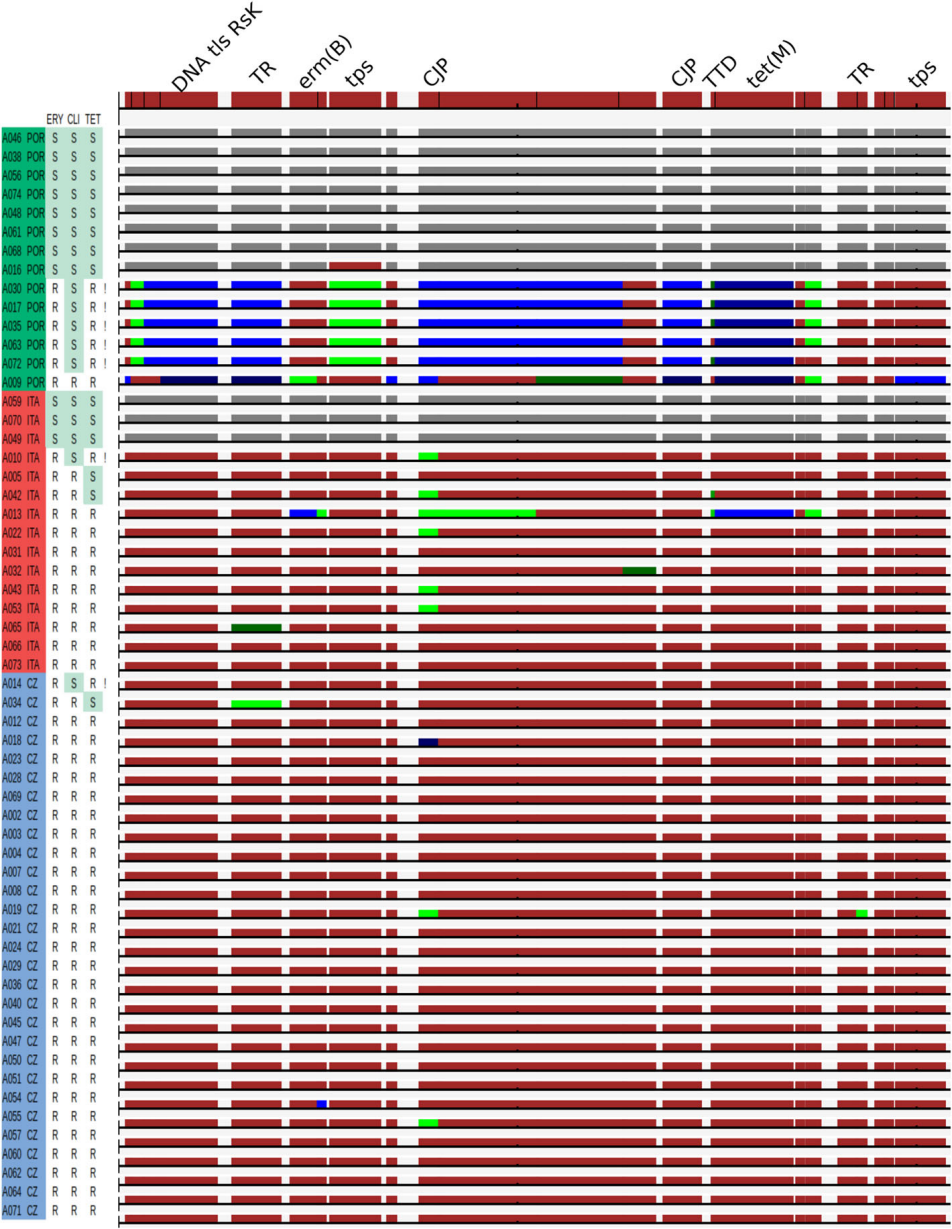
SNPs within genes presented in Tn*6002* transposon. Genes with sequences identical between a particular strain and the corresponding gene in the Tn*6002* are coloured in dark brown. Genes with sequences differing to reference in one SNP same for more samples are coloured in shades of green, these differing in more SNPs same for more samples in shades of blue. Genes which were not found in particular genomes are coloured in grey. Country of origin and resistance (R, resistant; S, susceptible) are indicated alongside the strain identifiers. Inducible clindamycin resistance is marked with “!” in the last column. Labelled are genes associated with resistance and gene transfer. (TR, transcriptional regulator; CJG, conjugal transfer protein; TTD, tetracycline resistance determinant leader peptide; tps, transposase; tls, translocase)

All genomes containing *erm*(B) and *tet(*M) carried all genes constituting Tn*6002* as well. These were identical to reference in 29 of 47 isolates (Fig. 4.). They differed from reference either in 1–2 separate genes (11 isolates) or in most of genes (7 isolates). The later were apparently carried by three different mobile genetics elements.

## Discussion

After introduction of PCV7, serotype 19A ranked globally among the top non-PCV7 serotypes associated with serotype replacement [26–29]. Molecular analysis of 19A post-PCV7 isolates revealed, that majority of isolates belong to 3 clonal complexes: CC199, CC320/271, and CC695 [26]. While CC320/271 arose by a serotype switch from ancestral ST’s 271 and 236 of serotype 19F, representing a “vaccine-escape” event, those representing CC695 arose from recombination involving both non-PCV7 and PCV7 serotypes since the ST695 serotype 19A originated from a penicillin susceptible ST695 serotype 4 and a penicillin intermediate ST199 serotype 19A [30]. It was suggested that the observed capsular switches were mainly driven by selective pressure of antibiotic consumption, as altered *pbp* genes associated with penicillin non-susceptibility have been identified in isolates belonging to those clones. Clonal complex CC199, represented by ST199, is widely geographically distributed as penicillin non-susceptible serotype 19A or penicillin susceptible serotype 15B/C [31, 32].

Likely, penicillin susceptible isolates of serotype 15B/C served as progenitor for penicillin non-susceptible ST199 serotype 19A. Study of serotype switches in CC199 in isolates from pre-PCV13 era in Germany identified at least two serotype switches from 19A to 15B of ST199, suggesting that capsular switches in ST199 is not unusual [33].

All assemblies of sequences surrounding *cps* locus presented in the study confirm serotype switch, although used methods do not allow to identify position, and orientation of genes. Nevertheless, the high variability of particular sequences indicates that these areas are changing very quickly within the clone and could be associated with multiple serotype switch events.

The results indicate that there is a recent common ancestor between the Netherlands^15B^-37 representative and the isolates included in the present study. This conjecture is supported by the SNP phylogeny and by the match in the sequences associated with the *cps* locus. The specific evidence is that the entire 15B *cps* locus present in the Netherlands^15B^-37 between the *pbp*2x and *pbp*1a genes was replaced in the studied isolates by the 19A *cps* locus. It is worthy of note that the engBF sequence situated downstream the locus is 100% identical in most of the isolates within the collection (with individual SNPs in three isolates). The engBF sequence consists of two parts: the first part (the base pairs 1.1309) is the same in all studied isolates whilst showing 97.6% identity to the corresponding sequence in Netherlands^15B^-37 representative and the second part (the remaining base pairs after 1309) are 100% identical to Netherlands^15B^-37. It is very likely that the present isolates share the beginning of the sequence with the conjectured donor of the 19A *cps* locus. If this is the case, the a*li*A gene, situated between the *eng*BF and *cps* locus, should also originate from this donor. It is remarkable that whilst the sequence of a*li*A is quite variable within the entire studied collection there is a substantial group (a majority) of isolates that have the a*li*A sequence 100% identical to the Netherlands^15B^-37 representative. This observation suggests that, when the conjectured serotype switch occurred, the donor had the *ali*A alleles identical to the Netherlands^15B^-37 representative. A similar situation is apparent upstream of the *cps* locus, where the *dex*B gene originates probably from the same donor. It is very likely that the serotype switch took place once and included the sequences surrounding the *cps* locus, but not the penicillin binding proteins. In addition to the common donor it seems that the genomes of a few of the isolates included in the present study were changed by recombinations in different genes afterwards. It is possible that some isolates, especially those which phylogenically differ from rest of the collection, originate from different serotype switches. Nevertheless the sequences where the exchange actually happened (*eng*F and *dex*B) are identical within the present collection and it is unlikely that they would originate from distinct serotype switches.

An increase of serotype 19A after introduction of PCV’s (PCV10 and PCV13) in the Czech Republic was due to an expansion of ST416 which is a DLV of ST199. It was proposed that an emergence of this subclone was facilitated by the presence of pilus 1 (PI-1), as group founder ST199 was found to be PI-1 negative [22]. The ST416 was identified in other European countries (Italy, Portugal, Germany), but it’s contribution to the increase of serotype 19A varies [5, 7, 8]. Antibiotic consumption may enhance the spread of certain drug-resistant clones. It is a plausible explanation that increased use of macrolides favoured the spread of this macrolide-resistant clone. Despite a decrease of macrolide consumption in Italy and Portugal during last decade, consumption of macrolides is high in both countries, and it shows a significant increasing trend in Czech Republic [34].

The isolates in the study represent two different forms of genetic diversification within ST416. Topology of the tree (Fig. 1) suggests that the strains present in Clade II possibly reflect and old lineage that has had time to diversify. The Clade I on the other hand, consists of strains with very similar core genomes although originating in different countries within a relatively long timescale, suggesting the rapid spread of a highly successful lineage that seems to be adapting mostly through recombination.

Recombination events led to the alteration of sequence type and it was followed by acquisition of a transposable elements carrying *erm*(B) and *tet*(M) genes in particular subclones and to variability within accessory genes. Sequences associated with transposable elements were not successfully assembled, because de-novo assembly frequently fails in repetitive sequences. Aligning reads to representative sequences of genes of interest gives a good overview of variability within isolates but does not inform about positioning and orientation of genes, not even about gene compositions of a given mobile element and accurate identification of transposable elements. It is also the reason, why inducible resistance to clindamycin in 7 isolates carrying *erm*(B) gene cannot be explained in this study, as it is associated with inverted repeat sequences located in the region upstream the gene [35]. Gene *erm*(B) is usually associated with constitutive resistance to clindamycin, but inducible resistance was also reported [36]. Isolates resistant to erythromycin belonging to ST199 were reported to carry *mef* genes [37], however *mef* genes were not present in the genome of any isolate in the study, neither in the genome of Netherlands^15B^-37.

## Conclusions

The elimination of vaccine serotypes through the use of PCVs promotes the spread of pre-existing minor clones as well as of novel clones arising from genetic exchange. Using whole genome sequencing we described the properties of ST416 serotype 19A, and the relationship between ST416 and ST199. Our study shows that ST416 developed from the Netherlands^15B^-37 clone and that it can now be found in several European countries. Comparing to the ancestral ST199 genotype, isolates of ST416 have additional accessory genes coding for antibiotic resistance. Whereas the capsular switch apparently featured only once, high variability of sequences in accessory genes confirms repeated horizontal gene transfer of mobile genetic elements carrying drug resistant genes.

## Methods

We used a collection of 58 isolates of serotype 19A of ST416 recovered between 2002 to 2016 in the Czech Republic, Italy, and Portugal to analyse their sequence diversity, and to investigate whether the spread of ST416 could be related to transfer of genes associated with antibiotic resistance.

### Bacterial isolates

Altogether, 58 isolates of *Streptococcus pneumoniae* of serotype 19A of ST416 were analysed (Additional file 1). Pneumococcal isolates (*n* = 29) were collected by the National Reference Laboratory for Antibiotics (National Institute of Public Health, Czech Republic) from 2010 to 2015, 15 isolates were obtained from the Department of Infectious Diseases (National Institute of Health, Rome, Italy) between 2002 to 2016 and 14 isolates were collected by the Faculdade de Medicina (Universidade de Lisboa, Lisbon, Portugal) between 2002 and 2011. The majority (*n* = 50; 86.2%) of isolates was collected from blood (*n* = 47) or cerebrospinal fluid (*n* = 3), Suppl. Table 1. The isolates were characterised by MLST, serotyping was performed by Quellung reaction as previously described [5, 7, 8]. MICs of penicillin, cefotaxime, erythromycin, clindamycin, tetracycline, chloramphenicol, trimethoprim/sulfamethoxazole were determined by broth microdilution method according to the EUCAST recommendations [38].

### WGS analysis

All isolates presented in the study were sequenced in the Laboratory of Antibiotic Resistance and applications of Mass Spectrometry in Microbiology (Charles University, Faculty of Medicine in Pilsen, Biomedical Centre Pilsen) using the Illumina MiSeq platform for paired end reads. Reads were trimmed by Trimmomatic v. 0.36 [39]. Trimmed reads were assembled de novo by SPAdes v 3.9.1 [40] and the resulting contigs were annotated with PROKKA v 1.12 [41]. De novo assemblies were uploaded to PubMLST [24] for confirmation of sequence types. PubMLST was also used for estimation of relatedness between sequence types 416 and 199.

Core genes of collection consisting of genomes under study and that of Netherlands^15B^-37 were set by Orthofinder v. 2.0.0 [42]. Coding sequences of orthologs were used to set core genome multilocus sequence typing (cgMLST) scheme for the collection with BLAST v. 2.2.31. Minimum spanning tree was constructed with Bionumeric v. 7.6.

Trimmed reads were aligned to particular reference sequences (Netherlands^15B^-37, 19A *cps* locus, Tn*6002*, *erm*(A), *erm(*TR)*,* and *mef*A/*mel* gene) using SMALT (SMALT is Copyright (C) 2010–2015 Genome Research Ltd). Consensus sequences were reconstructed using SAMtools [43] and VCFtools [44]. Only unambiguously calling bases with minimum values for DP (depth) > 4, MQ (mapping quality) > 50, and QUAL (position quality) > 50 were accepted.

Consensus sequences obtained by mapping to reference strain Netherlands^15B^-37 (ATCC BAA-1663) were used for construction of a phylogeny tree based on single nucleotide polymorphisms (SNP). The reference sequence was obtained by concatenation of contig sequences downloaded from GenBank (BioSample: SAMN00792697). SNP phylogeny based on the Netherlands^15B^-37 reference enabled elimination of the sites involved in recombination and that resulted in more accurately branching tree. Recombination sequences were predicted on the basis of the SNPs densities in the sliding window of 101 sites in length. The cut-off was set for each sequence separately as ratio of number of SNPs and length of sequence. Sites where recombination emerged at least in one genome were cut out from all the genomes. RaxML in default setting, which calculates ICA and consequently TCA scores, was used to generate unrooted tree with model GAMMA. Lengths (L) of branches correspond to probability that mutation occurs on a site, e.g. two nodes differ in every 1/Lth site.

Sequences positioned between genes coding for penicillin binding proteins (*pbp*), *pbp*2x and *pbp*1a were obtained by mapping reads to corresponding sequences in the genome of Netherlads^15B^-37 (*pbp*2x – *dex*B, *ali*A – *pbp*1a), and to the sequence of *S. pneumoniae* serotype 19A capsular locus (accession number JF9111520). Consensus sequences were inspected manually. Differences between sequences coding particular genes situated between *pbp*2x and *pbp*1a in the genome of reference sequence, and in the genome of every isolate, were described according to number of variants within collection and the origin of change (point mutation or recombination, Fig. 2b) [25].

Presence of particular genes coding for resistance to antibiotics (macrolides, tetracycline) was determined by mapping reads to sequences of *mef*(A)/*mel* (accession number EF042094.1), *mef*(E) (accession number U83667.1), *erm(*A) (accession number KT803896848263.1), *erm(*TR) (accession number AF002716) and to the transposon Tn*6002* (accession number AY898750.1) which included genes *erm(*B), and *tet(*M).

## Supplementary information

**Additional file 1. **List of *Streptococcus pneumoniae* clinical isolates included in the study. Table contains data of origin and properties of the isolates. (CZ, the Czech Republic; ITA, Italy; POR, Portugal; F, female; M, male; PEN, penicillin; CTX, cefotaxime; ERY, erythromycin; CLI, clindamycin; TET, tetracycline; CMP, chloramphenicol; SXT, trimethoprim/sulfamethoxazole; Induction, inducible clindamycin resistance).

## Data Availability

Whole genome sequencing data are available in the NCBI Short Read Archive under BioProject accession PRJNA600380.

## Abbreviations

CC: Clonal complex
cgMLST: core genome multilocus sequence typing
*cps*: caplular polysaccharide
DLV: Double-locus variant
IPD: Invasive pneumococcal disease
MIC: Minimal inhibitory concentration
MLST: Multilocus sequence typing
PCV: Pneumococcal conjugate vaccines
SNP: Single nucleotide polymorphism
ST: Sequence type
